# Acquisition and expression of conditioned taste aversion differentially affects extracellular signal regulated kinase and glutamate receptor phosphorylation in rat prefrontal cortex and nucleus accumbens

**DOI:** 10.3389/fnbeh.2014.00153

**Published:** 2014-05-07

**Authors:** Roberto Marotta, Sandro Fenu, Simona Scheggi, Stefania Vinci, Michela Rosas, Andrea Falqui, Carla Gambarana, M. Graziella De Montis, Elio Acquas

**Affiliations:** ^1^EM Laboratory, Department of Nanochemistry, Istituto Italiano di Tecnologia - IITGenova, Italy; ^2^Department of Toxicology, University of CagliariCagliari, Italy; ^3^Centre of Excellence on Neurobiology of Addiction, University of CagliariCagliari, Italy; ^4^National Institute of Neuroscience - INN, University of CagliariCagliari, Italy; ^5^Department of Neuroscience, University of SienaSiena, Italy; ^6^Department of Life and Environmental Sciences, University of CagliariCagliari, Italy

**Keywords:** conditioned taste aversion (CTA), extracellular signal regulated kinase (ERK), glutamate receptors, immuno electron microscopy, nucleus accumbens (Acb), prefrontal cortex (PFCx)

## Abstract

Conditioned taste aversion (CTA) can be applied to study associative learning and its relevant underpinning molecular mechanisms in discrete brain regions. The present study examined, by immunohistochemistry and immunocytochemistry, the effects of acquisition and expression of lithium-induced CTA on activated Extracellular signal Regulated Kinase (p-ERK) in the prefrontal cortex (PFCx) and nucleus accumbens (Acb) of male Sprague-Dawley rats. The study also examined, by immunoblotting, whether acquisition and expression of lithium-induced CTA resulted in modified levels of phosphorylation of glutamate receptor subunits (NR1 and GluR1) and Thr^34^- and Thr^75-Dopamine-and-cAMP-Regulated^ PhosphoProtein (DARPP-32). CTA acquisition was associated with an increase of p-ERK-positive neurons and phosphorylated NR1 receptor subunit (p-NR1) in the PFCx, whereas p-GluR1, p-Thr^34^- and p-Thr^75^-DARPP-32 levels were not changed in this brain region. CTA expression increased the number of p-ERK-positive neurons in the shell (AcbSh) and core (AcbC) but left unmodified p-NR1, p-GluR1, p-Thr^34^- and p-Thr^75-DARPP-32^ levels. Furthermore, post-embedding immunogold quantitative analysis in AcbSh revealed that CTA expression significantly increased nuclear p-ERK immunostaining as well as p-ERK-labeled axo-spinous contacts. Overall, these results indicate that ERK and NR1, but not GluR1 and DARPP-32, are differentially phosphorylated as a consequence of acquisition and expression of aversive associative learning. Moreover, these results confirm that CTA represents an useful approach to study the molecular basis of associative learning in rats and suggest the involvement of ERK cascade in learning-associated synaptic plasticity.

## Introduction

Conditioned taste aversion (CTA) is a rapid to establish, robust, and long-lasting experimental model of associative learning based on animals' ability to avoid a taste (conditioned stimulus, CS) that has been previously associated with visceral malaise (unconditioned stimulus, US) (Garcia et al., 1955; Scott, 2011). In order to develop, CTA requires that a gustatory short-term memory trace is translated into the formation of long-term memory, i.e., gene expression (Berman et al., 1998) and into long-term molecular modifications (McGaugh, 2000) which can also be recapitulated as synaptic plasticity (Shiflett and Balleine, 2011). Accordingly, CTA has offered, over the years, a valuable tool to investigate, in rodents, the neurotransmitter receptors (Fenu et al., 2001, 2005, 2009; Fenu and Di Chiara, 2003; Akirav, 2007; Barki-Harrington et al., 2009) involved in aversive associative learning, its underpinning molecular mechanisms (Berman et al., 1998; Yasoshima et al., 2006; Bernstein and Koh, 2007; Kwon and Houpt, 2012) as well as its anatomical substrates (Dunn and Everitt, 1988; Yamamoto et al., 1995; Berman et al., 1998; Ferreira et al., 2006; Ramírez-Lugo et al., 2007; Elkobi et al., 2008; Kwon and Houpt, 2012). In this regard, the analysis of neurochemical, molecular and neuroanatomical mechanisms involved in distinct phases (acquisition, consolidation, and expression) of CTA has long represented a significant approach to untangle their significance in aversive associative learning (Scott, 2011).

A large number of pharmacological studies, based on the use of receptor agonists and antagonists, supports the critical role that dopamine (DA) (Hoffman and Beninger, 1988; Fenu et al., 2001, 2005, 2009; Fenu and Di Chiara, 2003; Cannon et al., 2005) and glutamate (Yasoshima et al., 2000; Akirav, 2007; Simonyi et al., 2009; Núñez-Jaramillo et al., 2012), in particular, play in different brain regions and in distinct phases of CTA. Accordingly, DA and glutamate have been found to be concomitantly released in the insular cortex while memory consolidation is in progress and blockade of DA D_1_ and glutamate NMDA receptors, in this cortex, results in prevention of memory formation (Guzman-Ramos et al., 2010). Similarly, in agreement with the observation that DA D_1_ receptor antagonists prevent aversive learning (Acquas et al., 1989; Acquas and Di Chiara, 1994; Fenu et al., 2001), blockade of DA D_1_ receptors by systemic SCH 23390, as well as by local administration of SCH 39166 in the shell of the nucleus accumbens (AcbSh), impairs CTA learning (Fenu et al., 2001). Conversely, the DA D_1_ and D_2_ receptor agonists, SKF38393 and quinpirole, respectively, have been shown to potentiate CTA acquisition (Hoffman and Beninger, 1988) and these effects are also mimicked by the indirect DA agonist d-amphetamine (Greenshaw and Buresová, 1982; Fenu and Di Chiara, 2003; Fenu et al., 2010). On the other hand, the role of glutamate transmission in the facilitation of the enduring changes underlying memory trace formation is supported by studies with NMDA receptor blockade (Rosenblum et al., 1997) and with NMDA receptor knock-out mice (Cui et al., 2005).

Among the molecular substrates worth of investigation in distinct phases of CTA, one of the most relevant and highly related to DA D_1_ and glutamate NMDA receptor activation is Extracellular signal Regulated Kinase (ERK) (Girault et al., 2007; Shiflett and Balleine, 2011), a kinase deeply involved in mechanisms of signaling at the basis of synaptic plasticity (Fasano and Brambilla, 2011). In one of the first studies aimed to address the role of ERK activation during acquisition of an aversively conditioned response to a taste, it was demonstrated that its activation takes place after long- but not short-term taste memory in the insular cortex (Berman et al., 1998), a key area involved in processing of gustatory information (Dunn and Everitt, 1988). Furthermore, the ability of distinct phases of CTA to activate ERK phosphorylation has been investigated in a number of other brain regions including the amygdala (Languille et al., 2009; Lin et al., 2010), the hippocampus (Languille et al., 2009) and the prefrontal cortex (PFCx) (Mickley et al., 2005; Lin et al., 2010) and overall these data point to complex and differential brain region-, phase- and time-dependent patterns of ERK activation.

The PFCx and the Acb, because of their anatomical connections (Groenewegen et al., 1990), are viewed as brain areas relevant for associative memory formation, retention, and extinction under distinct aversive and fear learning tasks such as CTA (Fenu et al., 2001; Mickley et al., 2005; Ferreira et al., 2006; Yasoshima et al., 2006; Ramírez-Lugo et al., 2007; Lin et al., 2010) and trace fear conditioning (Runyan and Dash, 2004; Gilmartin and Helmstetter, 2010). In CTA experiments, in particular, the role of both PFCx (Mickley et al., 2005; Yasoshima et al., 2006; Lin et al., 2010) and Acb (Fenu et al., 2001; Fenu and Di Chiara, 2003; Núñez-Jaramillo et al., 2012) has been investigated but the involvement of ERK phosphorylation has only been addressed, to the best of our knowledge, in the PFCx during CTA extinction (Lin et al., 2010).

On the basis of these premises, the aim of the present study was to assess whether acquisition and expression of lithium-induced CTA would result in a differential activation of ERK. This goal was accomplished by immunohistochemical experiments aimed at determining the changes of p-ERK-positive neurons counts in PFCx as well as in AcbSh and AcbC during CTA acquisition and expression. In addition, the analysis of PFCx and Acb molecular changes during these phases of CTA was extended to the assessment, by immunoblotting experiments, of the phosphorylation of glutamate receptor subunits (NR1 and GluR1) as well as of Thr^34^- and Thr^75^-Dopamine-and-cAMP-Regulated PhosphoProtein (DARPP-32). Finally, the study also aimed at assessing, by immuno electron microscopy, the subcellular (dendritic vs. somatic) distribution of ERK phosphorylation, as demonstrated in the immunohistochemical experiments, in the AcbSh of rats that expressed the acquired CTA.

## Materials and methods

### Animals

Male Sprague-Dawley rats (250–275 g) [Harlan Laboratories Srl, S. Pietro al Natisone (UD), Italy] were housed in groups of 4 per cage for at least 5 days before use and were maintained on a 12:00/12:00 h light/dark cycle (lights on at 8:00 a.m.) with food and water available *ad libitum*. Experiments were carried out between 10:00 a.m. and 2:00 p.m. All the experimental procedures were performed in accordance with the European and Italian legislation on the use and care of laboratory animals (EU Directive 2010/63 of September 22, 2010 and Italian D.L. 27.01 1992, n. 116). All efforts were made to minimize the number of animals used and their suffering.

### Behavioral experiments

Behavioral experiments consisted of three phases [training, acquisition (conditioning) and expression (testing)] and lasted up to 8 days. In training, acquisition and expression phases the volumes of tap water and fresh sucrose solution drunk by rats were recorded.

#### Training

During this phase rats had to learn to consume their daily fluid intake in 20 min/day sessions. On the day before the beginning of training, rats were single-housed and the following day they were presented for 20 min with a graduated fluid reservoir filled with tap water after 24 h of water deprivation. Training took place along 6 days, i.e., until rats acquired a constant fluid intake, defined as at least three consecutive days in which fluid intake did not differ more than 10%. During training rats' body weight was monitored and found to be not lower than 80% of that of other rats (same age) of the colony that did not undergo fluid restriction.

#### Acquisition (Acq)

This phase followed the last day of training and lasted 1 day. During acquisition rats were presented a 15% (w/v) sucrose solution or water as CS for 20 min.

***Experiment 1 (Acq1)***. One hour after drinking sucrose solution (CS^+^) or water (CS^−^), rats were administered lithium chloride (LiCl, 125 mg/kg i.p., in isotonic −0.15 M- solution) as unconditioned stimulus (US^+^) or saline i.p. (US^−^). Rats of these four groups (i.e., CS^+^-US^+^; CS^−^-US^+^; CS^+^-US^−^; CS^−^-US^−^) were anesthetized and underwent transcardiac perfusion for p-ERK immunohistochemistry, 30 min after US presentation, in PFCx and Acb.

***Experiment 2 (Acq2)***. In order to control for the effect of fluid deprivation and scheduled drinking, rats of two distinct groups, one presented with sucrose (CS^+^), the other presented with water (CS^−^), were anesthetized and underwent transcardiac perfusion for p-ERK immunohistochemistry, 30 min after CS^+^ or CS^−^ presentation, in PFCx and Acb.

***Experiment 3 (Acq3)***. In order to avoid a possible confounding factor in CTA acquisition response, due to the modifications induced by sucrose (CS^+^) consumption on NR1, GluR1, and DARPP-32 phosphorylation pattern (Danielli et al., 2010), rats were sacrificed 3 h after US. In particular, four separate groups of rats, two lithium-conditioned (CS^+^-US^+^ and CS^−^-US^+^) and two control groups (CS^+^-US^−^ and CS^−^-US^−^) underwent a conditioning protocol with presentation of CS^+^ or CS^−^ and were sacrificed, 3 h after US^+^ or US^−^, for immunoblotting detection of NR1, GluR1, Thr^34^- and Thr^75^-DARPP-32 phosphorylation in PFCx and Acb.

#### Expression (Expr)

Expression tests took place 24 h after acquisition of CS-US association.

***Experiment 4 (Expr1)***. In order to detect changes of p-ERK expression by immunohistochemistry in PFCx and Acb, two groups of rats, lithium-conditioned (CS^+^-US^+^) and controls (CS^+^-US^−^), were presented the sucrose solution (CS^+^) for 20 min and 10 min later (i.e., 30 min after the beginning of CS presentation) were anesthetized to undergo transcardiac perfusion.

***Experiment 5 (Expr2)***. In the immunocytochemical experiments for p-ERK detection in AcbSh by immuno electron microscopy, two lithium-conditioned (CS^+^-US^+^) and two control groups (CS^+^-US^−^) were given distinct time intervals (3 and 23 min, indicated as groups A and B, respectively in results, figures and figures' legends) from the beginning of CS presentation, before anesthesia for transcardiac perfusion.

***Experiment 6 (Expr3)***. Finally, in order to assess the ability of CTA expression to affect NR1, GluR1 and Thr^34^- and Thr^75^-DARPP-32 phosphorylation, two groups of rats (CS^+^-US^+^ and CS^+^-US^−^) were presented, 24 h after CTA acquisition, the sucrose solution (CS^+^) for 20 min. An additional control group, never exposed to the acquisition phase (CS^−^-US^−^) was presented tap water for 20 min. Ten minutes after the test for CTA expression (i.e., 30 min after the beginning of CS^+^ or CS^−^ presentation) rats were sacrificed for western blotting analysis for detection of NR1, GluR1, Thr^34^- and Thr^75^-DARPP-32 phosphorylation in PFCx and Acb.

### Immunohistochemistry and immunocytochemistry

In CTA acquisition experiments (**Acq1** and **Acq2**), rats were anesthetized with chloral hydrate (450 mg/kg i.p.) 30 min after CS or US presentation. Similarly, in CTA expression experiments, rats were anesthetized 30 min **(Expr1)** for immunohistochemistry and 3 and 23 min (**Expr2**) for immunocytochemistry, after the beginning of expression test. Under deep anesthesia, rats from **Acq1**, **Acq2** and **Expr1** groups were subjected to transcardiac perfusion with ice-cold PBS (Phosphate Buffered Saline: 137 mM, 2.7 mM KCl, 10 mM Na_2_HPO_4_,2 mM KH_2_PO_4_, pH 7.4) and 4% paraformaldehyde (PFA). Similarly, under deep anesthesia, rats from **Expr2** groups, were subjected to transcardiac perfusion with ice-cold PBS (Phosphate Buffered Saline: 137 mM, 2.7 mM KCl, 10 mM Na_2_HPO_4_,2 mM KH_2_PO_4_, pH 7.4), ice cold 4% paraformaldehyde and 0.5% glutaraldehyde in 0.1 M cacodylate buffer for immuno electron microscopy processing. After perfusion, brains were removed and post-fixed overnight in the same fixative used for transcardiac perfusion.

#### Immunoperoxidase processing and quantitative analysis

Brain sections (40 μm) of the regions of interest, PFCx and Acb, were cut, according to the rat brain atlas of Paxinos and Watson (1998) on ice-cold PBS with a vibratome (Leica VT1000, Leica, Germany), kept in ice-cold PBS and processed for immunohistochemistry according to a protocol for free-floating sections. Immunoreactions for p-ERK-positive cells detection was applied to at least three serial slices obtained from each experimental subject. Slice processing and image analysis were performed as described in detail in our previous studies (Acquas et al., 2007; Ibba et al., 2009). Briefly, after incubation with 1% H_2_O_2_, sections were incubated with 3% Bovine Serum Albumin (BSA). The incubation with primary antibody [anti di-phosphorylated forms of ERK_1/2_ phospho-p44/42 MAPK ERK_1/2_, Cell Signaling Technology, MA, USA (1:300)] was conducted overnight. On the following day, after rinsing, sections were incubated for 1 h with the biotinylated secondary antibody (1:800) and after further rinses the sections were incubated in avidin biotin peroxidase complex prepared according to the manufacturer's suggestions (Vectastain ABC kit, Vector Laboratories, CA, USA) and a 3–3'-diaminobenzidine solution (10 mg/ml) was added until development of brown staining. Sections were mounted onto gelatine-coated slides and processed through alcohol-xylene for light microscopy examination. p-ERK-positive neurons were identified at the lowest magnification (10X) and quantitative analysis was performed using a Zeiss Axioskop 40 light microscope, equipped with PL Floutar 10X (*na* = 0.3), 40X (*na* = 1.00–0.5) and 100X oil (*na* = 1.3) objectives, coupled with a digital camera (PixeLink, PL-A686C, 6.6 MPixels). Images of the regions of interest were obtained at the lowest magnification (10X) according to the rat brain atlas of Paxinos and Watson (1998) and used to automatically count the number of p-ERK-positive neurons/area (density/area) by application of ImageJ in conjunction with automated background subtraction, to avoid experimenter bias, and entropy threshold plug-in (Kapur et al., 1985).

#### Immuno electron microscopy and quantitative analysis

Coronal vibratome sections (100 μm) were cut on ice-cold PBS with a vibratome (Leica VT1000 P, Leica, Germany). Discs of tissue (1.2 mm in diameter) corresponding to a portion of the AcbSh, were high-pressure frozen (Leica EM PACT2) and freeze substituted (Leica EM AFS2) in anhydrous methanol containing 0.5% uranyl acetate (modified from van Lookeren Campagne et al., 1991). The samples were finally embedded at −45°C in Lowicryl HM20 under UV lights. Thin sections (~ 70 nm thick) obtained on a Leica EM UC6 were collected on Formvar coated nickel grids.

Post-embedding immunolabeling was performed according to Mathiisen et al. (2006): briefly, sections were washed with 0.1% sodium borohydride and 50 mM glycine in Tris-buffered saline containing 0.1% Triton X-100 (TBST), blocked in TBST containing 2% BSA, and then incubated with the primary antibody. We used two rabbit polyclonal antibodies against respectively the un-phosphorylated and the di-phosphorylated forms of ERK_1/2_ (p44/42 MAPK ERK_1/2_ and phospho-p44/42 MAPK ERK_1/2_ at Thr202/Tyr204, Cell Signaling Technology, MA, USA) diluted 1:50 in the same blocking solution for 2.5 h at room temperature. The samples were subsequently washed with TBST and incubated with goat anti-rabbit Ig coupled to 10 nm gold particles diluted 1:100 in the same blocking solution for 1 h. The sections were then washed repeatedly in TBS, post-fixed in 1% glutaraldehyde in TBS for 5 min, washed with distilled water, and finally stained with uranyl acetate and lead citrate. The grids were observed in a Jeol JEM 1011 electron microscope operating at 100 KV and recorded with an 4 Mp Gatan Orius SC100 Charge-Coupled Device (CCD) camera. To test method specificity in the immunocytochemical procedures the ERK and p-ERK antibodies were omitted and under these conditions no immunoreactivity was observed. Moreover, p-ERK immunostaining signal was abolished when the samples grids were incubated for 1 h with a mixture of p-ERK and the corresponding blocking peptide for phospho-p44/42 (1:2, Phospho-p44/42 MAPK ERK_1/2_ Thr202/Tyr204, Blocking Peptide #1150, Cell Signaling Technology, MA, USA).

The density of p-ERK nuclear and cytoplasmic labeling (expressed as number of gold particles/μm^2^) was calculated on a total of eleven animals (five controls, and six lithium-conditioned rats from the CTA expression groups (**Expr2**). More than 30 neuron cell bodies from each animal, belonging to at least two different sections were analyzed and imaged in non-overlapping electron micrographs at a final magnification of 5545. This led to a total of more than 300 neuron cell bodies analyzed, corresponding to nuclear and cytoplasmic areas of, respectively, more than 19,000 and 8000 μm^2^. The relative number of p-ERK-positive axo-spinous synaptic contacts, and their density, were counted in the same animals. We randomly sampled a total of 4630 asymmetric synapses, 1046 of which were p-ERK labeled in either pre- or post-synaptic profile. The axo-spinous synaptic contacts were imaged in non-overlapping electron micrographs at a final magnification of 5545. This led to an average of more than 400 asymmetric synapses analyzed in each animal, 96 of which were p-ERK positive. Axo-spinous synaptic contacts were identified for the presence of both synaptic vesicles and post-synaptic density (PSD) respectively in pre- and post-synaptic terminals. To determine the sub-synaptic distribution of p-ERK in dendritic spine profiles the spine head profile was subdivided into three distinct compartments: core, shell, and PSD (Rácz et al., 2004; Boggio et al., 2007). The number of gold particles and their association with each of these compartments were evaluated in a total of more than 200 p-ERK-positive axo-spinous synaptic contacts, from both lithium-conditioned and control rats. Labeling values were expressed as the density of gold particles found in each spine compartment.

#### Immunoblotting detection of NR1, GluR1, Thr^34^- and Thr^75^-DARPP-32 phosphorylation and quantitative analysis

Rats were sacrificed, their heads were briefly immersed (3–5 s) in liquid nitrogen, and the brains were rapidly removed and cut into 1 mm slices using an ice-cold metal brain matrix (ASI Instruments, Inc., MI, USA). The PFCx and Acb were quickly dissected out from the slices corresponding to plates 7–9 and 10–12, respectively, of the rat brain atlas (Paxinos and Watson, 1998). Tissues were flash-frozen in liquid nitrogen and stored at −80°C until assayed. Immunoblotting was performed as previously described (Scheggi et al., 2007; Danielli et al., 2010) and was carried out with phosphorylation-state-specific antibodies against p-Ser897 NR1and p-Ser845 GluR1, (Santa Cruz Biotechnology, CA, USA) and against p-Thr^34^ DARPP-32 and p-Thr^75^ DARPP-32 (Cell Signaling Technology, MA, USA), or antibodies not phosphorylation state-specific against total NR1 and GluR1 (Santa Cruz Biotechnology, USA) or total DARPP-32 (Cell Signaling Technology, MA, USA). Membranes were then incubated with peroxidase-conjugated affinity-purified secondary antibodies (Pierce Biotechnology Inc., IL, USA). Antibody binding was detected using a chemiluminescence detection system (Pierce Biotechnology Inc. IL, USA) and quantified with the Versa Doc 1000 Imaging System (Bio-Rad Laboratories, CA, USA). Samples containing the same amount of total proteins from rats in each experimental group were run on the same immunoblots and then analyzed together. In the regions studied, the total amount of DARPP-32 was unmodified in the different experimental groups compared to the control group (data not shown). For each experiment, values obtained from experimental groups were calculated as the percentage of their respective control values.

### Drugs

Lithium Chloride (LiCl) (Sigma Aldrich, Italy) and Chloral hydrate (Sigma Aldrich, Italy) were dissolved in saline (NaCl 0.9%) and administered intraperitoneally (i.p.) at the doses, respectively, of 125 mg/kg/2 ml and 450 mg/kg/5 ml. D-Sucrose (Sigma Aldrich, Italy) solutions (15% w/v), made in tap water, were freshly prepared on the day of usage.

### Statistics

Fluid intake was expressed as average + s.e.m. of sucrose solution (ml) drunk by rats belonging to the same group. Average + s.e.m. of p-ERK-positive neurons/area (% p-ERK-positive neurons/area) were calculated as % change with respect to the average number of p-ERK-positive neurons/area of the control groups, set as 100%. These averages were used for statistical comparisons by Analyses of Variance (ANOVAs, Prism 4.0a, GraphPad Software Inc., CA, USA) which were applied to assess the statistical significance of first-order effects of multiple categorical factors. Least significant differences (LSD) *post-hoc* analyses, whereby allowed by ANOVAs, were applied for multiple comparisons, with the statistical significance set at *p* < 0.05. Data from electron microscopy experiments were analyzed by One-Way ANOVA with Origin 8 software (OriginLab Co., MA, USA). Statistical analyses of Western blot data were performed with Prism 4.0a, (GraphPad Software Inc., CA, USA); data were analyzed using ANOVA and *post-hoc* analysis was performed by Bonferroni's test, when *p* < 0.05, unless otherwise specified.

## Results

### Effect of saline or lithium chloride administration on sucrose intake (CTA expression)

**Figure 1:**
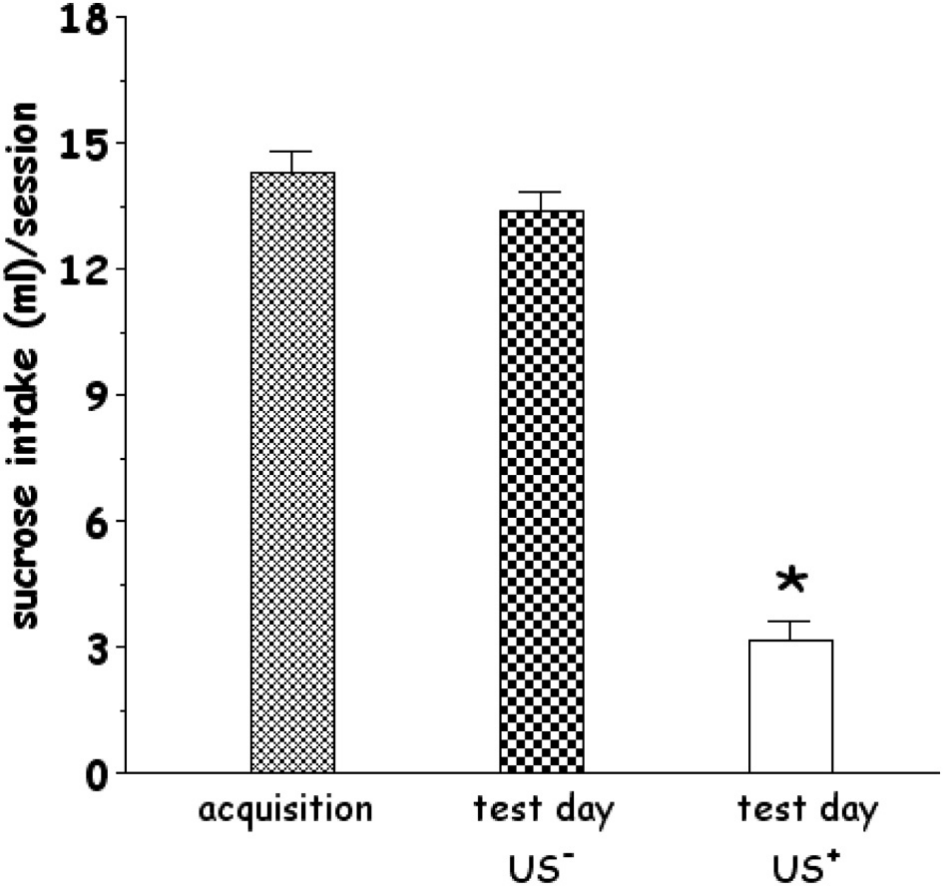
**Effect of systemic administration of lithium chloride (LiCl, 125 mg/kg i.p., CS^+^, or saline i.p., CS^−^) on average sucrose intake**. CS^+^ or CS^−^ were administered 1 h after the 20 min drinking session of the last training day (acquisition). Each histogram (*n* = 13 in each group) represents the average + s.e.m. of sucrose solution intake (ml) in 20 min. ^*^p < 0.05 vs. US^−^ (One-Way ANOVA followed by LSD *post-hoc* test).

As shown in Figure 1, rats consumed significantly different volumes of sucrose solution on acquisition and test days [One-Way ANOVA, *F*_(2, 36)_ = 157.95, *p* < 0.001]. Control rats (CS^+^-US^−^) drank in the expression test a similar volume (13.38 + 0.47 ml/test) compared with average sucrose intake drunk by rats on acquisition day (14.31 + 0.51 ml/test). In contrast, rats administered LiCl (CS^+^-US^+^) drank significantly less (3.15 + 0.49 ml/test) than controls. LSD *post-hoc* analysis revealed that CS^+^-US^+^ resulted in a significant reduction of sucrose intake compared to CS^+^-US^−^ group.

### Effect of CTA acquisition and expression on ERK phosphorylation: immunohistochemistry experiments

**Figure 2:**
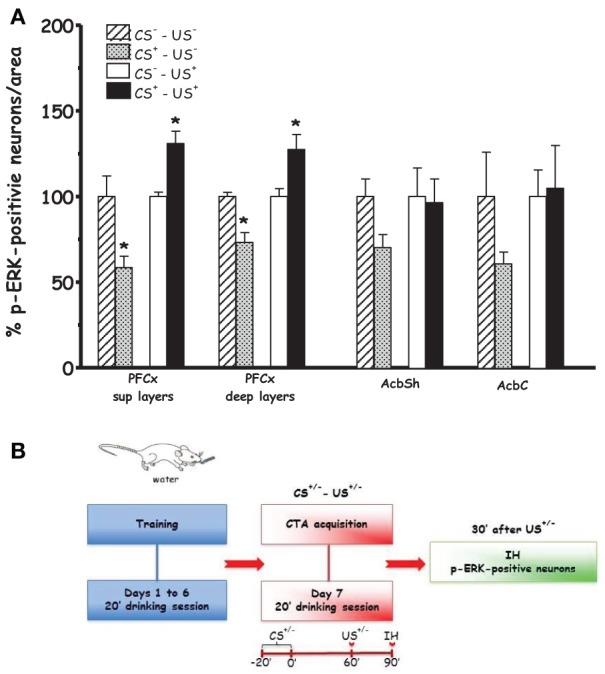
**Acquisition (Acq1) (A):** Effect of CTA acquisition on % changes of p-ERK-positive neurons/area in the PFCx (superficial and deep layers) and Acb (shell and core). Data, average + s.e.m., are expressed as % changes with respect to the effect in control groups (CS^−^) set as 100%. ^*^Indicates significant differences (*p* < 0.05) of % changes of p-ERK-positive neurons with respect to control groups (CS^−^-US^−^ and CS^−^-US^+^) (One-Way ANOVA followed by LSD *post-hoc* test) (*n* = 4 in each group); **(B):** timeline schedule of **Acq1** experiment.

Figures 2 and 5 show the effect of acquisition of CTA on % p-ERK-positive neurons in PFCx and Acb (**Acq1**). One-Way ANOVA revealed a significant effect of CS^+^ in both superficial and deep layers of PFCx ([*F*_(sup)1, 8_ = 9.14, *p* < 0.02] and [*F*_(deep)1, 8_ = 6.97, *p* < 0.03], respectively). In addition, One-Way ANOVA provided significant treatment effects (US^+^ exposure) in PFCx (superficial [*F*_(sup)1, 6_ = 16.28, *p* < 0.006] and deep [*F*_(deep)1, 6_ = 7.91, *p* < 0.03] layers), but not in AcbSh and AcbC ([*F*_(AcbSh)1, 6_ = 0.02, *p* < 0.87] and [*F*_(AcbC)1, 6_ = 0.03, *p* < 0.86], respectively). LSD *post-hoc* analysis revealed that 90 min after CS^+^ exposure p-ERK-positive neurons were significantly decreased with respect to CS^−^ exposure. Moreover, CS^+^-US^+^ association significantly increased % p-ERK-positive neurons in the superficial and deep layers of the PFCx with respect to CS^−^-US^+^ association.

**Figure 3:**
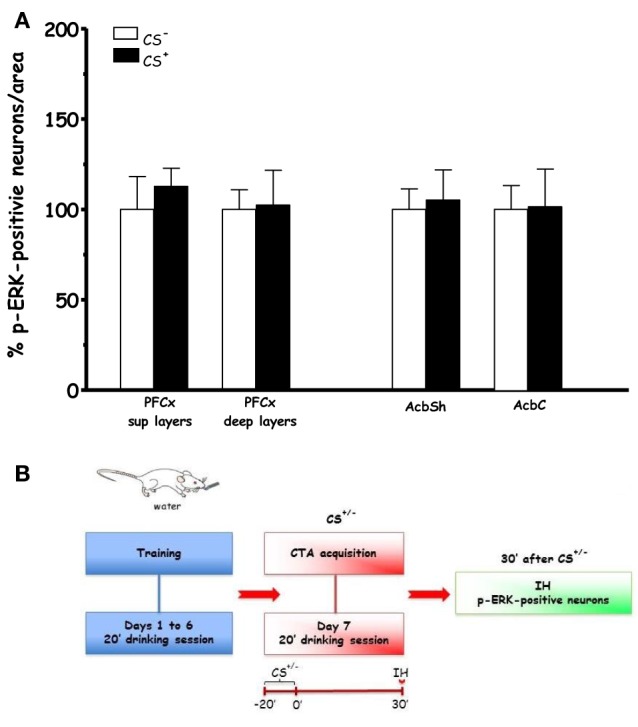
**Acquisition (Acq2) (A):** Effect of water (CS^−^) or sucrose (CS^+^) intake on % changes of p-ERK-positive neurons/area in the PFCx (superficial and deep layers) and Acb (shell and core). Data, average + s.e.m., are expressed as % changes with respect to the effect in control group (CS^−^) set as 100% (*n* = 4 in each group); **(B):** timeline schedule of **Acq2** experiment.

Figure 3 shows the effect on % p-ERK-positive neurons in PFCx and Acb after scheduled drinking of either sucrose solution (CS^+^) or tap water (CS^−^) during acquisition (**Acq2**). Under these conditions, ANOVA revealed that drinking sucrose failed to result in significant main effects in PFCx [*F*_(sup)1, 6_ = 0.6, *p* < 0.46] and [*F*_(deep)1, 6_ = 0.01, *p* < 0.91] and Acb ([*F*_(AcbSh)1, 6_ = 0.006, *p* < 0.80] and [*F*_(AcbC)1, 6_ = 0.003, *p* < 0.95]), suggesting that the effect found in the PFCx of rats in **Acq1** experiment were attributable to learning the CS-US association.

**Figure 4:**
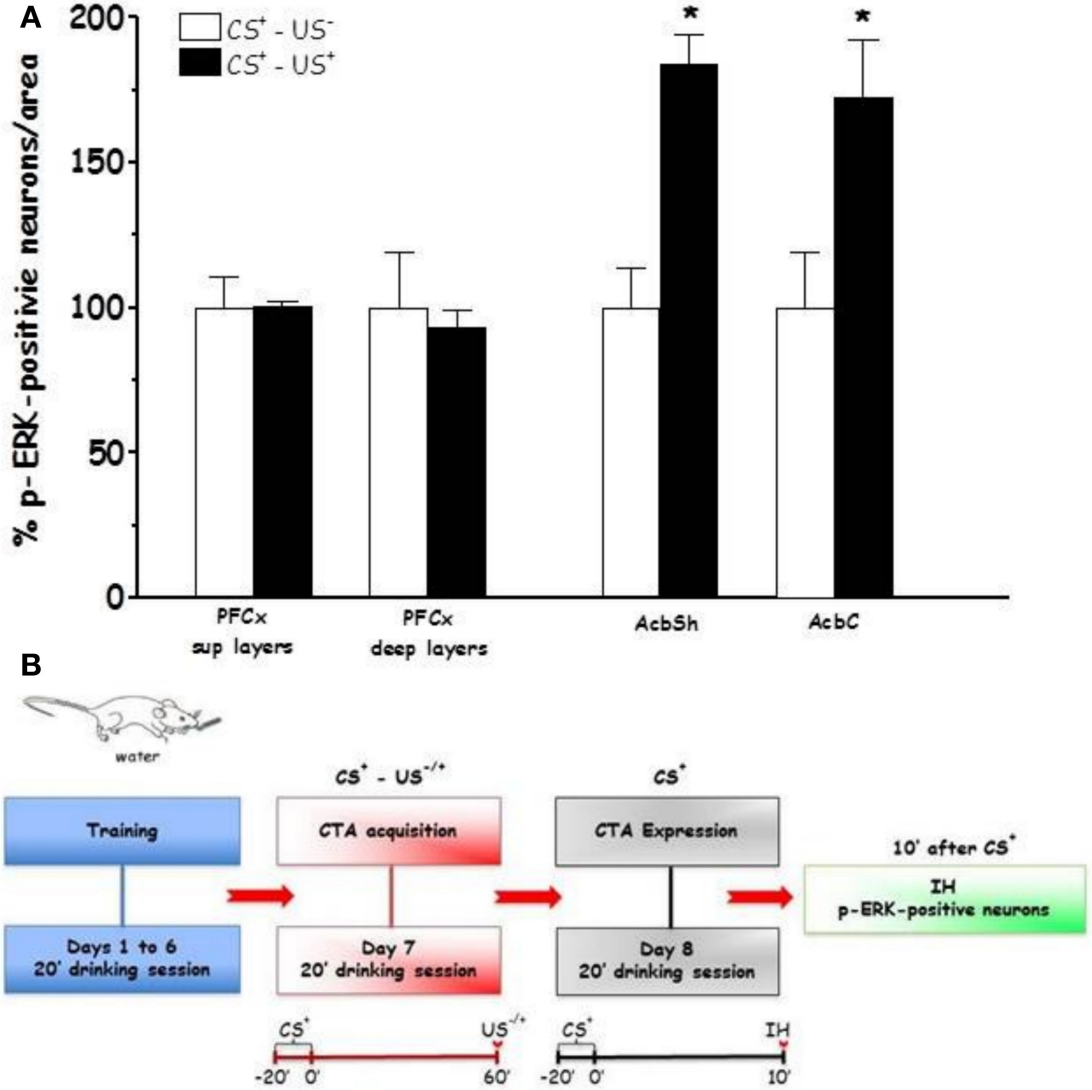
**Expression (Expr1) (A):** Effect of CTA expression on % changes of p-ERK-positive neurons/area in the PFCx (superficial and deep layers) and Acb (shell and core). Data, average + s.e.m., are expressed as % changes with respect to the effect in control group (CS^+^-US^−^) set as 100%. Lithium chloride (LiCl, 125 mg/kg i.p., US^+^) or saline (i.p., US^−^) were administered on the last training day 1 h after the drinking session. Rats were sacrificed 30 min after the beginning of the expression test. ^*^Indicates significant differences (*p* < 0.05) of % changes of p-ERK-positive neurons with respect to controls (CS^+^-US^−^) (One-Way ANOVA followed by LSD *post-hoc* test) (*n* = 4 in each group); **(B):** timeline schedule of **Expr1** experiment.

**Figure 5:**
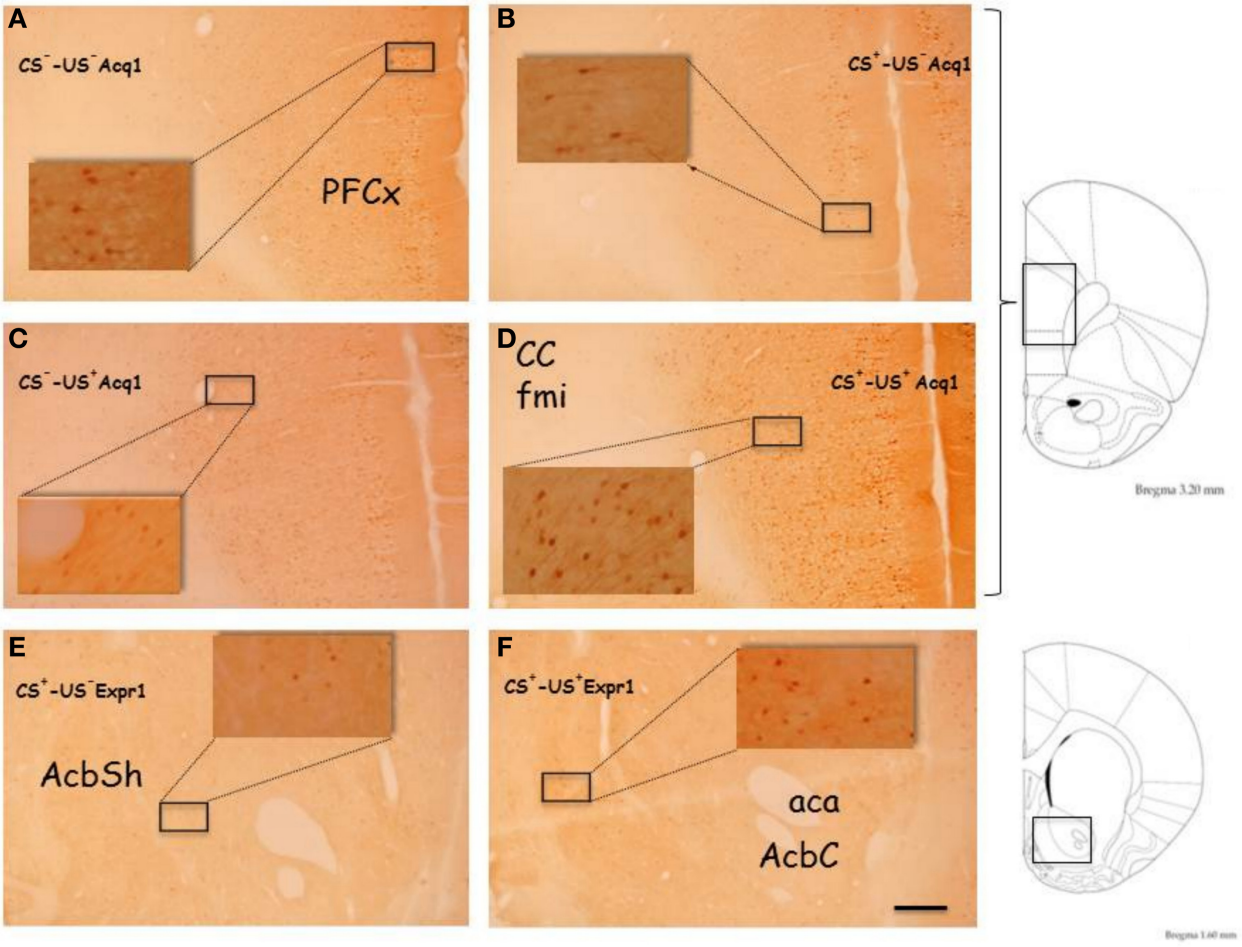
**Representative, low (5X) and high (40X, inserts) magnification images of p-ERK immunostaining from PFCx of CS^−^-US^−^ (A, Acq1) and CS^+^-US^−^ rats (B, Acq1), fom PFCx of CS^−^-US^+^ (C, Acq1) and CS^+^-US^+^ rats (D, Acq1) and from Acb of CS^+^-US^−^ (E, Expr1) and CS^+^-US^+^ rats (F, Expr1)**. The images on the right are taken from Paxinos and Watson (1998) rat brain atlas and indicate the antero-posterior gradient (mm) used to cut the brains and to select the slices for immunostaining. Abbreviations: aca, anterior commissure, anterior part; AcbSh, Accumbens Shell; AcbC, Accumbens Core; CC fmi, forceps minor of the corpus callosum; PFCx, Prefrontal cortex. Scale bar indicates 200 μm in low magnification images.

Figures 4 and 5 show the effect of CTA expression on % p-ERK-positive neurons in rats (CS^+^-US^−^ and CS^+^-US^+^ groups) sacrificed 10 min after completion of CTA test (**Expr1**). One-Way ANOVA revealed significant effects of conditioning in AcbSh and AcbC ([*F*_(AcbSh)1, 6_ = 8.99, *p* < 0.024] and [*F*_(AcbC)1, 6_ = 6.84, *p* < 0.039], respectively). In contrast, One-Way ANOVA failed to reveal significant effects of conditioning on % changes of p-ERK-positive neurons in PFCx superficial and deep layers ([*F*_(sup)1, 6_ = 0.0002, *p* < 0.98] and [*F*_(deep)1, 6_ = 0.12, *p* < 0.73], respectively). LSD *post-hoc* analysis revealed that expression of LiCl-induced CTA increased ERK phosphorylation in AcbSh and AcbC.

### Effect of CTA expression on subcellular ERK phosphorylation distribution in AcbSh: immuno electron microscopy experiments

**Figure 6:**
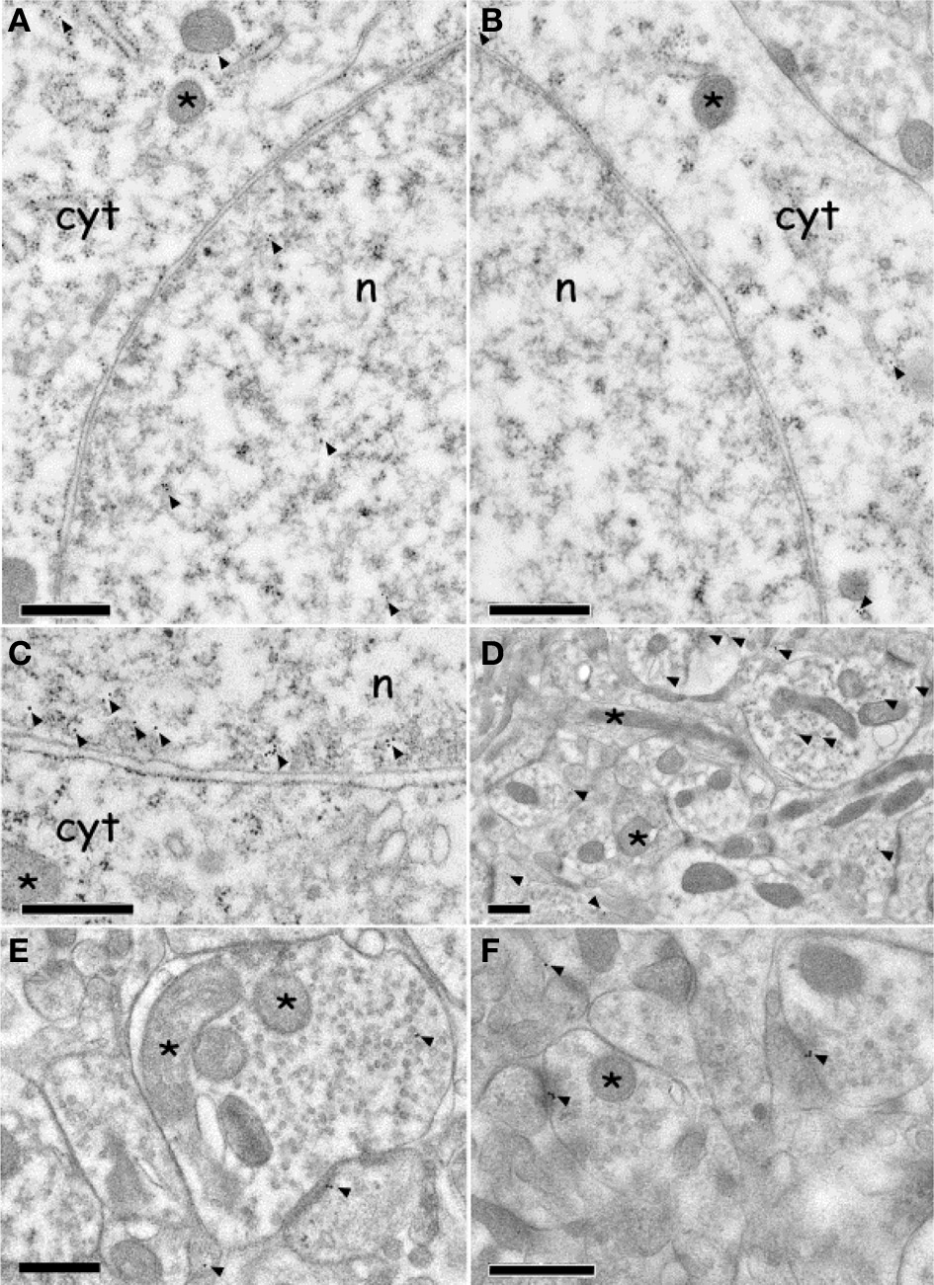
**Immuno electron microscopy localization of ERK and p-ERK inside the AcbSh neurons of (CS^+^-US^+^) rats (Expr2). (A,C):** p-ERK immunolabeling in neuron cell body nucleus and cytoplasm (arrowheads). The asterisks indicate mitochondria. **(B):** ERK immunolabeling in neuron cell body cytoplasm (arrowheads). The asterisks indicate mitochondria. **(D–F):** p-ERK immunolabeling in dendrites, dendritic spines, and pre-synaptic boutons (arrowheads). Asterisks indicate mitochondria. Abbreviations: cyt, cytoplasm; n, nucleus. Scale bars are 0.5 μm.

Neuron cell bodies p-ERK signal was mainly expressed in the nucleus, and to a lesser extent in the cytoplasm (Figures 6A and C); on the contrary the signal of un-phosphorylated ERK was expressed exclusively inside the cytoplasm (Figure 6B). Nuclear p-ERK signal was often present in clusters of 2–4 gold particles, frequently close to the nuclear membrane (Figure 6C).

**Figure 7:**
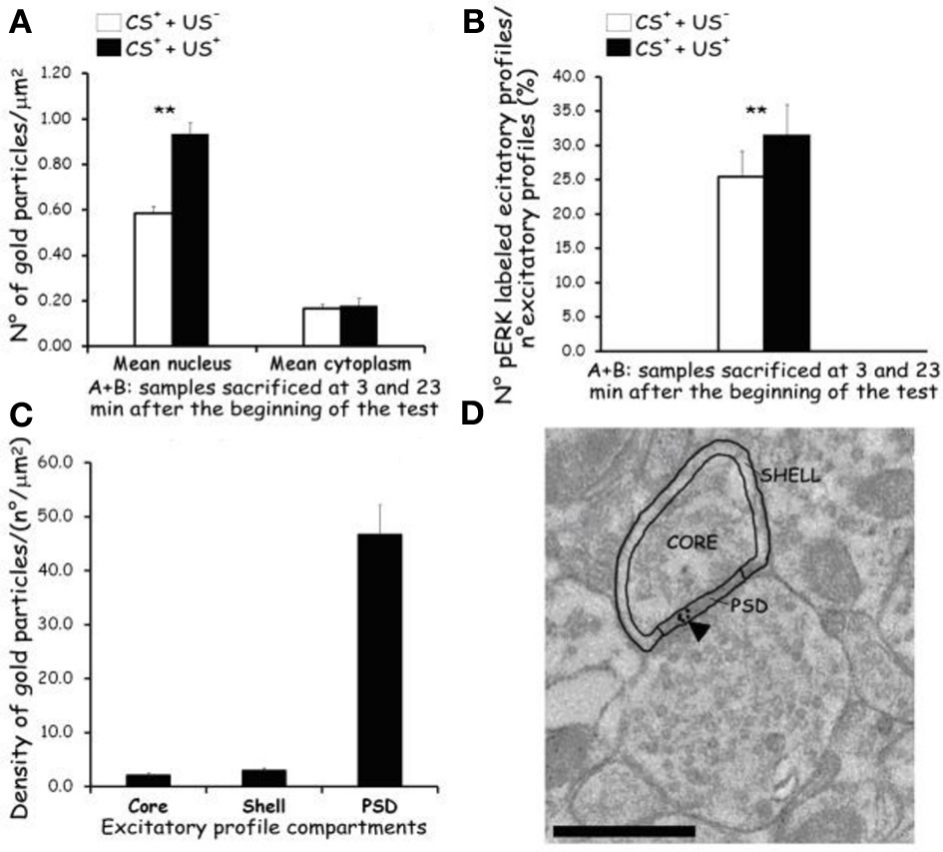
**Effects of CTA expression on subcellular p-ERK distribution. (A):** histograms showing p-ERK density inside nucleus and cytoplasm of neuron cell bodies belonging to AcbSh of lithium conditioned (CS^+^-US^+^) and control rats (CS^+^-US^−^) (**Expr2**); histograms represent pooled data obtained from rats sacrificed 3 (group **A**) and 23 (group **B**) min after the beginning of testing. Values are average + s.e.m. ^**^Indicate significant differences (*p* < 0.01) in average density gold particles between US^−^ and US^+^ rats (One-Way ANOVA). **(B):** histograms showing the percentage of p-ERK-positive synaptic profiles out of the total number of synapses that were randomly sampled inside the AcbSh of lithium conditioned (CS^+^-US^+^) and control rats (CS^+^-US^−^) (**Expr2**); histograms represent pooled data obtained from rats sacrificed 3 (group **A**) and 23 (group **B**) min after the beginning of testing. Values are average + s.e.m. ^**^Indicate significant differences (*p* < 0.01) in number of pERK labeled excitatory profiles (%) between US^−^ and US^+^ rats (One-Way ANOVA). **(C,D):** sub-synaptic distribution of p-ERK in dendritic spine compartments (core, shell, and PSD). **(C):** histograms showing the density of gold particles (number of gold particles/μm^2^) observed in each sub-synaptic compartment. **(D):** Representative TEM micrograph of a dendritic spine subdivided in three compartments: core, shell, and PSD. Arrowhead points to gold particles. Scale bar is 500 nm.

The average density of p-ERK nuclear labeling in AcbSh neurons of lithium-conditioned (CS^+^-US^+^) rats did not differ between animals sacrificed 3 or 23 min from beginning of testing (expression groups A and B) and thus data were pooled. Average density of p-ERK nuclear labeling in AcbSh neurons of lithium-conditioned (CS^+^-US^+^) rats was significantly higher than in control rats (CS^+^-US^−^) (0.93 ± 0.05 vs. 0.58 ± 0.03 gold particles/μm^2^; Figure 7A and Table 1, One-Way ANOVA, *p* < 0.01). Cytoplasmic p-ERK signal was significantly lower than nuclear density (One-Way ANOVA, *p* < 0.01) and not significantly different between lithium-conditioned and control rats (Figure 7A and Table 1).

**Table 1:** **Phospho-ERK average density and percent of p-ERK-labeled excitatory profiles inside the AcbSh of lithium treated (CS^+^-US^+^) and control rats (CS^+^-US^−^) obtained pooling the results from rats sacrificed at 3 and 23 min after the beginning of test**.

**CS^+^ - **US**^−^**		**CS^+^ - **US**^+^**	
A + B: samples sacrificed at 3 and 23min after start of expression test		A + B: samples sacrificed at 3 and 23min after start of expression test	
**p-ERK average density**		**p-ERK average density**	
Nuclear	Cytoplasmic	Nuclear	Cytoplasmic
Mean ± s.e.m.	Mean ± s.e.m.	Mean ± s.e.m.	Mean ± s.e.m.
0.58 ± 0.03	0.17 ± 0.02	0.93 ± 0.05^**^	0.18 ± 0.04
**% p-ERK-labeled excitatory profiles**		**% p-ERK-labeled excitatory profiles**	
25.5 ± 3.7		31.6 ± 4.4^**^	

pERK positive synaptic profiles values are expressed as percentage of p-ERK-labeled excitatory profiles out of the total number of excitatory profiles;

** Indicate respectively significant differences (p < 0.01) of average density gold particles and number of pERK labeled excitatory profiles (%) between US^−^ and US^+^ rats (One-Way ANOVA).

p-ERK immunoreactivity in the AcbSh medium spiny neurons, during CTA expression, was not restricted to the cell body, but extended to dendritic and axonal processes (Figures 6D–F). Accordingly, p-ERK labeling was detected in dendritic spines, mainly at synaptic plasma membrane domains, but also in pre-synaptic axonal boutons forming connections with dendritic spines (Figures 6D–F). The number of p-ERK labeled axo-spinous contacts was higher in the AcbSh of lithium treated rats (CS^+^-US^+^) with respect to controls (CS^+^-US^−^) (31.6 ± 4.4 vs. 25.5 ± 3.7 out of the total number of p-ERK-labeled excitatory profiles; expression groups A + B; One-Way ANOVA, *p* = 0.01) (Figure 7B and Table 1).

We also investigated the distribution of p-ERK immunoreactivity within AcbSh dendritic spines in both lithium-conditioned (CS^+^-US^+^) and control (CS^+^-US^−^) rats from A + B expression groups. In both groups a large majority of p-ERK immunoreactivity was expressed inside PSD whereas only a minority of gold nanoparticles was associated with shell and core of spines' head (Figures 7C and D).

### Effect of CTA acquisition and expression on Nr1, GluR1, Thr^34^-, and Thr^75^-DARPP-32 phosphorylation levels: immunoblotting experiments

For the immunoblotting experiments rats of CTA acquisition experiment 3 (**Acq3**) were sacrificed 3 h after the last treatment (US) in order to avoid a possible confounding factor in CTA acquisition response, due to the modifications induced by sucrose (CS) consumption on NR1, GluR1, Thr^34^- and Thr^75^-DARPP-32, phosphorylation patterns (Danielli et al., 2010). The phosphorylation levels of NR1, GluR1, Thr^34^ and Thr^75^ DARPP-32 were assessed in PFCx, AcbSh, and AcbC and the results are shown in Table 2. Statistical analysis of phosphorylation levels of the NR1 and GluR1 subunits of NMDA and AMPA glutamate receptors indicated a difference between groups in the levels of p-NR1 in PFCx [*F*_(3, 19)_ = 8.19; *p* < 0.01]. Data analysis by One-Way ANOVA indicated no significant difference among p-Thr^34^ and p-Thr^75^ DARPP-32 levels in all 3 regions of the four experimental groups (Table 2). Bonferroni's multiple comparison test revealed that p-NR1 levels were higher in the group of rats that acquired CTA than in other groups (*p* < 0.01) (Table 2). No statistically significant differences were found in the levels of p-NR1 in AcbSh and AcbC. Moreover, no statistical differences were found in the levels of p-GluR1 in PFCx nor Acb. In the CTA expression experiment 6 (**Expr3**), rats were sacrificed 10 min after being presented the sucrose solution (CS) for 20 min (i.e., 30 min after the beginning of CS presentation). The phosphorylation pattern of NR1 and GluR1 subunits and Thr^34^ and Thr^75^ DARPP-32, was measured after testing for CTA expression (Table 3). One-Way ANOVA of the results indicated no significant differences among the levels of phosphoproteins in PFCx, AcbSh, and AcbC of these experimental groups.

**Table 2:** **Levels of p-NR1, p-GluR1 p-Thr^34^ and p-Thr^75^ DARPP-32, in different brain regions after CTA acquisition**.

**Groups**	**% of PhosphoProtein levels**			
	**p-NR1**	**p-GluR1**	**p-Thr^**34**^ DARPP-32**	**p-Thr^**75**^ DARPP-32**
***PFCx***				
CS^−^-US^−^	100	100	100	100
CS^+^-US^+^	136.7 ± 9.4^**^	115.3 ± 9.6	106.6 ± 4.1	104.9 ± 1.7
CS^+^-US^−^	104.2 ± 3.2	101.2 ± 6.2	103.8 ± 6.4	105.2 ± 5.6
CS^−^-US^+^	103.4 ± 4.0	97.8 ± 4.7	98.1 ± 6.9	100.7 ± 5.6
***AcbSh***				
CS^−^-US^−^	100	100	100	100
CS^+^-US^+^	108.2 ± 4.3	104.7 ± 2.0	98.8 ± 23.4	140.1 ± 29.8
CS^+^-US^−^	105.4 ± 5.2	102.5 ± 4.9	105.7 ± 14.0	114.6 ± 7.8
CS^−^-US^+^	104.2 ± 5.4	104.7 ± 5.4	87.4 ± 10.5	117.9 ± 24.2
***AcbC***				
CS^−^-US^−^	100	100	100	100
CS^+^-US^+^	124.2 ± 9.3	109.8 ± 7.6	82.8 ± 7.0	98.7 ± 13.4
CS^+^-US^−^	101.4 ± 5.1	94.6 ± 10.6	92.4 ± 4.7	85.9 ± 12.7
CS^−^-US^+^	101.2 ± 7.1	100.2 ± 1.0	112.7 ± 10.5	75.1 ± 12.6
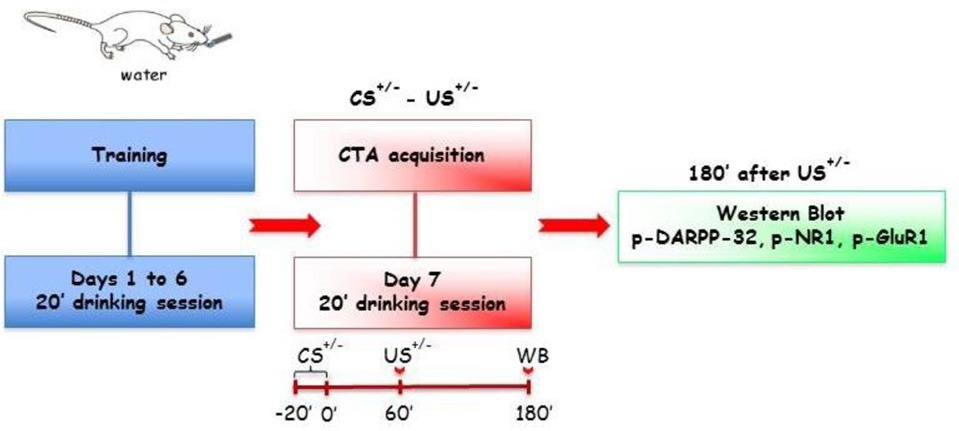				

Values are the average ± s.e.m. percentage of values in saline treated (CS^−^) rats. Figure depicts timeline schedule of **Acq3** experiment.

** Indicates p < 0.01 with respect to CS^−^-US^−^.

**Table 3:** **Levels of p-NR1, p-GluR1, p-Thr^34^ and p-Thr^75^ DARPP-32, in different brain regions after CTA expression**.

**Groups**	**% of PhosphoProtein levels**			
	**p-NR1**	**p-GluR1**	**p-Thr^**34**^ DARPP-32**	**p-Thr^**75**^ DARPP-32**
***PFCx***				
CS^−^-US^−^ Test: Water	100	100	100	100
CS^+^-US^+^ Test: CS^+^	109.3 ± 7.4	104.8 ± 5.2	102.3 ± 5.5	98.1 ± 3.2
CS^+^-US^−^ Test: CS^+^	114.2 ± 3.9	108.8 ± 6.5	99.3 ± 654	99.4 ± 2.2
***AcbSh***				
CS^−^-US^−^ Test: Water	100	100	100	100
CS^+^-US^+^ Test: CS^+^	108.2 ± 6.4	97.5 ± 6.5	106.6 ± 21.7	100.4 ± 12.4
CS^+^-US^−^ Test: CS^+^	122.5 ± 7.2	111.7 ± 9.1	164.6 ± 38.5	121.2 ± 18.9
***AcbC***				
CS^−^-US^−^ Test: Water	100	100	100	100
CS^+^-US^+^ Test: CS^+^	100.2 ± 3.3	102.8 ± 3.1	108.4 ± 11.1	100.8 ± 20.9
CS^+^-US^−^ Test: CS^+^	110.4 ± 6.9	102.0 ± 1.8	148.1 ± 19.1	89.5 ± 17.6
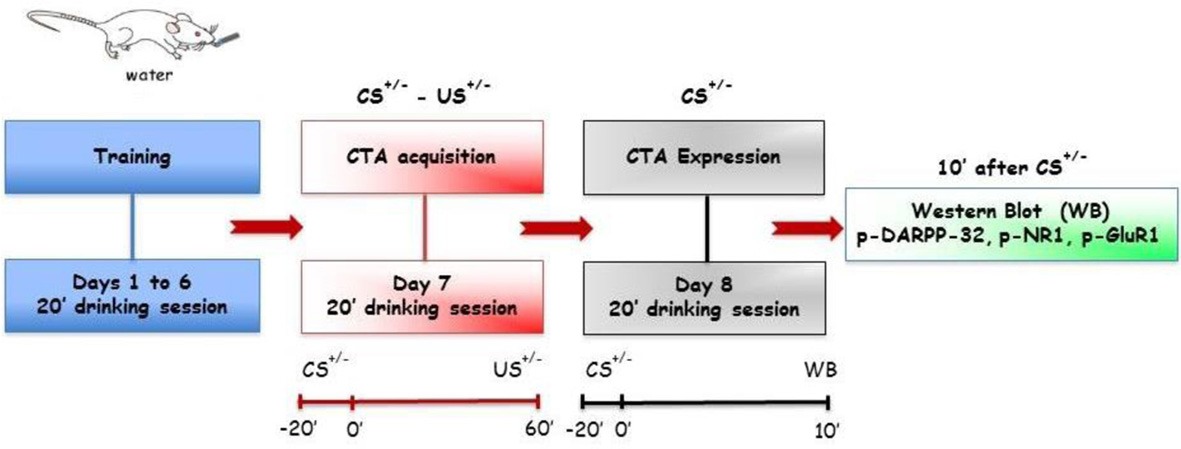				

Values are the average ± s.e.m. percentage of values in saline treated (CS^−^) rats. Figure depicts timeline schedule of **Expr3** experiment.

## Discussion

The results of the present study demonstrate that acquisition and expression of lithium-induced CTA results in differential phosphorylation of ERK and NR1 subunit of NMDA glutamate receptor in rats' PFCx and Acb. In particular, acquisition of lithium-induced CTA resulted in increased phosphorylation of ERK and NR1 in the PFCx while, expression of lithium-induced CTA disclosed an increase of ERK phosphorylation in AcbSh and AcbC. Furthermore, immuno electron microscopy analysis of the compartmentalization of p-ERK signal within the AcbSh of rats from CTA expression group revealed a significant increase of nuclear p-ERK immunostaining as well as a significantly higher number of p-ERK labeled axo-spinous contacts, mostly co-localized with PSD, with respect to control rats.

The study was also aimed to assess whether or not acquisition and expression of lithium-induced CTA would result in changes of phosphorylation levels of GluR1 AMPA glutamate receptor subunit and DARPP-32 in the same brain regions. The results revealed that, at least at the time intervals and experimental conditions used, both acquisition and expression of CTA were not accompanied by modifications in the phosphorylation of these substrates.

In agreement with previous studies from our (Fenu et al., 2001; Fenu and Di Chiara, 2003) and other laboratories (Yasoshima et al., 2006), we found that a single CS-US association reliably induced CTA (Figure 1). Thus, the observed effects of CTA acquisition and expression on ERK and NR1 phosphorylation can be considered behaviorally relevant, i.e., phase- and brain region-dependent. The immunohistochemical analysis revealed that the number of p-ERK-positive neurons/area was significantly reduced in the PFCx (both in the superficial and deep layers) 90 min after CS^+^exposure (i.e., 30 min after US^−^) This decrease in PFCx p-ERK-positive neurons may be part of the delayed response to a prolonged, 20 min, exposure to a palatable taste, while at an earlier time the number of p-ERK-positive neurons/area was not modified (Figure 3). This modification may be an expression of the complex phenomenon of neuronal plasticity in response to a relevant natural stimulus (a caloric palatable food) in an area, the PFCx, anatomically (Berendse and Groenewegen, 1990; Brog et al., 1993; Heidbreder and Groenewegen, 2003) and functionally (Cardinal et al., 2002) connected with the AcbSh, where short-term habituation to palatable taste is observed in dopaminergic transmission (Bassareo et al., 2002; Rauggi et al., 2005; Danielli et al., 2010). Notably, in both superficial and deep layers of PFCx CS^+^-US^+^ association increased, 30 min after US administration, the number of p-ERK-positive neurons/area (Figure 2). This effect could not be attributed to US or CS on their own. In particular, failure of US to elicit pERK expression in the PFCx is in agreement with previous studies showing that systemic lithium administration fails to affect ERK phosphorylation in mouse PFCx (Longoni et al., 2011); furthermore, the present results also indicate that sucrose consumption fails to affect ERK phosphorylation in PFCx (Figure 3) when measured 30 min after CS^+^ exposure, overall suggesting that increased ERK phosphorylation in PFCx accompanies the learning of the CS-US association. This conclusion appears in agreement with the observation that CTA acquisition was also accompanied by increased phosphorylation of NR1 receptor subunit in the same area. However, the modifications of ERK and NR1 receptor subunit phosphorylation observed in PFCx are temporarily distinct since assays for p-ERK, on one hand, and assays for p-NR1, p-GluR1, and p-DARPP-32, on the other hand, were conducted in slices obtained from rats sacrificed 30 min and 3 h, respectively, after US administration. As previously pointed out, such longer time interval for western blotting assay was selected in order to avoid possible confounding effects due to modifications in the phosphorylation levels of NR1, GluR1, and DARPP-32 in the PFCx of rats receiving sucrose solution as CS (Rauggi et al., 2005; Danielli et al., 2010). In fact, exposure to a palatable taste has been previously shown to result in increased NR1, GluR1, and Thr^34^-DARPP-32 phosphorylation in PFCx and Acb (Rauggi et al., 2005; Danielli et al., 2010). Hence, after a shorter time interval from CS, as in Experiment 1 (Acq1), we wouldn't have been able to dissociate between CS and US-CS association-elicited increases. Notably, sucrose consumption produces a DAergic response associated with transient modifications in NR1, GluR1 and DARPP-32 phosphorylation, in the PFCx, and Acb, that are dependent on the activation of a DA D_1_ receptor-PKA signaling pathway (Rauggi et al., 2005; Danielli et al., 2010). These modifications have been proposed to play a role in the formation of a gustatory memory trace (Danielli et al., 2010) and may represent the mechanism underpinning the increase in p-ERK positive neurons found in the PFCx 90 min after sucrose exposure and 30 min after US. Indeed, p-ERK levels are increased by activation of the DA D_1_ receptor-PKA signaling pathway or by NMDA transmission with different mechanisms, such as increased MEK activity or inhibition of STEP, a tyrosine phosphatase that inactivates ERK (Valjent et al., 2005; Nagai et al., 2007). The long-lasting increase in p-NR1 levels still present in the PFCx several hours after acquisition of CTA could represent a marker of sustained NMDA receptor activity. However, while our experimental design could not contribute to disclose any possible mechanistic and temporal relationship between ERK and NR1 receptor subunit phosphorylation (Xia et al., 1996; Hardingham et al., 2001), these results point to the PFCx as an anatomical site where neuronal plasticity takes place during and shortly after CTA acquisition (Gilmartin and Helmstetter, 2010). This possibility appears in agreement with the observation that time-dependent ERK phosphorylation in PFCx also plays a critical role in the extinction of acquired CTA (Lin et al., 2010). On the other hand, phosphorylation of AMPA receptor GluR1 subunit and DARPP-32 (either at Thr^34^- and Thr^75^-) was unmodified, suggesting that long-lasting modifications in PFCx phosphorylation levels of these substrates are not involved in CTA acquisition or expression.

In agreement with previous studies reporting that the Acb plays a critical role in associative learning and, in particular in lithium-induced CTA (Fenu et al., 2001; Fenu and Di Chiara, 2003; Yasoshima et al., 2006; Ramírez-Lugo et al., 2007), we found that expression of lithium-induced CTA resulted in ERK activation in both the AcbSh and AcbC. Previous studies aimed at addressing the role of AcbSh and AcbC DA in CTA acquisition disclosed a critical role for DA D_1_ receptors in the AcbSh (Fenu et al., 2001). Notably, although a close relationship exists between activation of DA D_1_ receptors signaling and ERK phosphorylation (Girault et al., 2007), in this study we detected changes of ERK phosphorylation in both the AcbSh and AcbC only during CTA expression. This finding appears in agreement with two lines of evidence obtained upon memory retrieval of conditioned taste aversion: (i) the observation of increased c-FOS-like immunoreactivity in both AcbSh and AcbC (Yasoshima et al., 2006) and (ii) the observation of a greater signal intensity of manganese-enhanced functional magnetic resonance imaging (MEMRI) in AcbSh and AcbC of conditioned rats (Inui-Yamamoto et al., 2010). However, no modifications were observed in the CTA acquisition phase in these Acb sub-regions and, as previously mentioned, also no changes in the pattern of NR1, GluR1 nor DARPP-32 phosphorylation were detected in the AcbSh or AcbC. Thus, while there is no doubt that DA plays a critical role in associative learning (Hoffman and Beninger, 1988; Mark et al., 1991; Fenu et al., 2001; Di Chiara, 2002), the present results overall suggests the possibility that on one hand stimulation of AcbSh DA D_1_ receptors during CTA acquisition (Fenu et al., 2001) does not result in changes of ERK phosphorylation and, on the other hand, that increased ERK phosphorylation upon CTA expression may not necessarily involve DA D_1_ receptors. This interpretation appears consistent with the lack of evidence, to the best of our knowledge, in support of a role of DA D_1_ receptors in CTA expression and also with the observation that intraoral saccharin administration to rats undergone lithium-induced CTA was responsible of a significant decrease of DA transmission in medial Acb (Mark et al., 1991).

The present study also represents the first immunocytochemical analysis of ERK and p-ERK expression inside AcbSh neurons of rats undergone testing to assess CTA expression. We coupled post-embedding immunolabeling with high pressure freezing (HPF) of pre-fixed rat brain tissues and freeze substitution (FS) (Sosinsky et al., 2008). This procedure yielded better brain ultrastructure and preserved tissue antigenicity better than after conventional EM processing (Sosinsky et al., 2008), being therefore suitable for post-embedding immunogold labeling (Humbel and Schwartz, 1989; van Genderen et al., 1991; van Lookeren Campagne et al., 1991) and quantitative immunocytochemistry (Oprins et al., 1994). Under these conditions, the observed ERK and p-ERK subcellular localization was largely coherent with previous immunocytochemical analyses performed on other brain regions (Liu et al., 2005; Zhu et al., 2006; Boggio et al., 2007; Xiao et al., 2011). In accordance with these studies, we found p-ERK expression inside the nuclei of AcbSh medium size spiny neurons and, to a lesser extent, in perinuclear cytoplasm and dendritic processes, mainly at pre- and post-synaptic profiles. In addition, in accordance with the results of the immunohistochemical analysis showing an increase of p-ERK positive neurons following CTA expression, we observed a significant increase of nuclear p-ERK immunostaining in AcbSh neurons of lithium-conditioned rats. These results suggest that AcbSh neurons of lithium-conditioned rats may be transcriptionally activated during CTA expression, given the role that p-ERK plays in phosphorylating several transcription factors once translocated inside the nucleus (Sétáló et al., 2001). Also, the number of p-ERK labeled axo-spinous contacts in the AcbSh of lithium-conditioned rats was significantly higher than in controls suggesting that CTA expression may have evoked an ERK activation at asymmetric contacts. Thus, in the AcbSh p-ERK may play a role in synaptic activation during CTA expression, as it has been shown in rat visual cortex after visual stimulation (Boggio et al., 2007). Moreover, we observed that in AcbSh of both conditioned and control rats p-ERK was localized preferentially in correspondence of PSD. This result, although different from the findings reported by Boggio et al. (2007) in rat visual cortex, appears consistent with the identification of p-ERK immunoreactivity at some of the post-synaptic active zones in pre-Bötzinger complex interneurons (Liu et al., 2005) and might suggest a close correlation between synaptic transmission and intracellular signaling transduction. Intriguingly, a specific role for synaptic function has been suggested for ERK_2_ isoform that has been detected in the PSD95 fraction obtained from rat synaptosomes and synaptic plasma membrane (Suzuki et al., 1995, 1999).

The immunohistochemical results in the AcbSh suggest that similar complex subcellular and synaptic changes might also take place in the AcbC. However, in light of the distinct afferents to the Acb activated upon CTA retrieval (Inui et al., 2013) and in light of the anatomical and functional differences between AcbSh and AcbC, future experiments are warranted to provide further insights on the subcellular distribution of p-ERK signal as well as on its role and significance in this Acb sub-region upon CTA expression.

In conclusion, this study reveals differential increases of PFCx ERK and NR1 phosphorylation, during CTA acquisition, and AcbSh and AcbC ERK phosphorylation during CTA expression. In particular increased ERK phosphorylation in the AcbSh during CTA expression, involves mechanisms both at synaptic level and in the regulation of gene expression (nuclear activation) as already shown in other brain areas (Boggio et al., 2007; Sindreu et al., 2007). Finally, in light of the increase of dendritic spines density found in AcbSh and AcbC during CTA expression (Acquas et al., 2011), these data overall suggest that local activation of p-ERK at synaptic sites may represent a mechanism associated with synaptic plasticity.

### Conflict of interest statement

The authors declare that the research was conducted in the absence of any commercial or financial relationships that could be construed as a potential conflict of interest.
