# Supplementary material for: Acquisition and expression of conditioned taste aversion differentially affects extracellular signal regulated kinase and glutamate receptor phosphorylation in rat prefrontal cortex and nucleus accumbens
Source: Front Behav Neurosci. 2014 May 7;8:153. doi: 10.3389/fnbeh.2014.00153 (PMC4019857; doi:10.3389/fnbeh.2014.00153)
Supplement: Supplementary file 1 [file DataSheet2.DOCX]

**Figure EM supplementary**

**
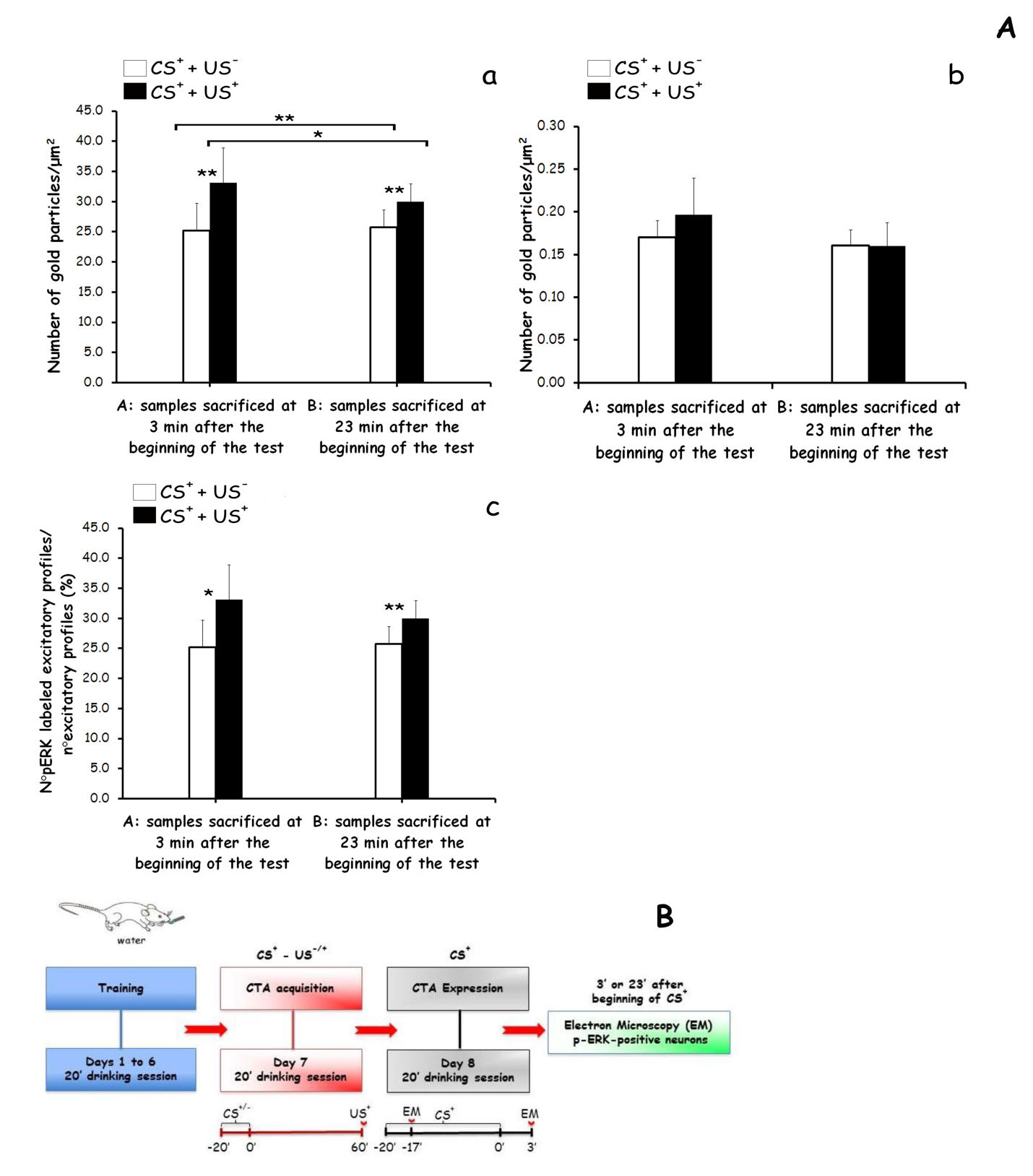
**

**Figure EM supplementary**. **(A):**  **a-b**: histograms showing p-ERK density inside nucleus (**a**) and cytoplasm (**b**) of neuron cell bodies belonging to AcbSh of lithium conditioned (CS**^+^-**US**^+^**) and control rats (CS**^+^-**US**^-^**) (expression group) sacrificed 3 (group A) and 23 (group B) minutes after the beginning of testing. **c**: histograms showing the percentage of p-ERK-positive synaptic profiles out of the total number of synapses randomly sampled from AcbSh of lithium-conditioned (CS**^+^-**US**^+^**) and control rats (CS**^+^-**US**^-^**) (expression group). Rats were sacrificed at 3 (group A) and 23 (group B) minutes after beginning of testing. Values are average + SEM. ***** and ****** indicate, respectively, significant differences (p<0.01 and *****p<0.05) of average density gold particles and number of pERK labeled excitatory profiles (%) (one-way ANOVA). **(B): timeline schedule of Expr2 experiment.**

**TABLE 1 supplementary**

| **CS^+^ + US^-^** | | | **CS^+^ + US^+^** | | |
| --- | --- | --- | --- | --- | --- |
| **Sacrifice at 3 minutes after start of expression test** | | | **Sacrifice at 3 min after start of expression test** | | |
| nuclear  average ± SEM | | cytoplasmic  average ± SEM | nuclear  average ± SEM | cytoplasmic  average ± SEM | |
| 0.56 ± 0.026 | | 0.17 ± 0.019 | 0.87 ± 0.039** | 0.20 ± 0.043 | |
| **% p-ERK-labeled excitatory profiles** | | | **% p-ERK-labeled excitatory profiles** | | |
| 25.2 ± 4.6 | | | 33.2 ± 5.8* | | |
| **Sacrifice at 23 minutes after start of expression test** | | | **Sacrifice at 23 minutes after start of expression test** | | |
| nuclear  average ± SEM | cytoplasmic  average ± SEM | | nuclear  average ± SEM | | cytoplasmic  average ± SEM |
| 0.61 ± 0.036 | 0.16 ± 0.019 | | 1.0 ± 0.06** | | 0.16 ± 0.027 |
| **% p-ERK-labeled excitatory profiles** | | | **% p-ERK-labeled excitatory profiles** | | |
| 25.7 ± 2.9 | | | 30 ± 3** | | |

**Table 1 supplementary**

Phospho-ERK average density and p-ERK-positive synaptic profiles inside the AcbSh of lithium treated (CS**^+^-**US**^+^**) and control rats (CS**^+^-**US**^-^**) sacrificed at 3 (group A) and 23 minutes (group B) after the beginning of test. p-ERK-positive synaptic profiles values are expressed as percentage of p-ERK-labeled excitatory profiles out of the total number of excitatory profiles; **^*^**Indicate significant differences (p<0.05) of number of pERK labeled excitatory profiles (%) between US**^-^** and US**^+^** rats (one-way ANOVA). **^**^**Indicate significant differences (p<0.01) in average density gold particles and number of pERK labeled excitatory profiles (%) between US**^-^** and US**^+^** rats (one-way ANOVA).
